# Unexpected Pro-Fibrotic Effect of MIF in Non-Alcoholic Steatohepatitis Is Linked to a Shift in NKT Cell Populations

**DOI:** 10.3390/cells10020252

**Published:** 2021-01-28

**Authors:** Daniel Heinrichs, Elisa F. Brandt, Petra Fischer, Janine Köhncke, Theresa H. Wirtz, Nurdan Guldiken, Sonja Djudjaj, Peter Boor, Daniela Kroy, Ralf Weiskirchen, Richard Bucala, Hermann E. Wasmuth, Pavel Strnad, Christian Trautwein, Jürgen Bernhagen, Marie-Luise Berres

**Affiliations:** 1 Department of Internal Medicine III, RWTH Aachen University, 52074 Aachen, Germany; d.heinrichs@fz-juelich.de (D.H.); ebrandt@ukaachen.de (E.F.B.); pfischer@ukaachen.de (P.F.); Janine.koehncke@web.de (J.K.); thwirtz@ukaachen.de (T.H.W.); ngueldiken@ukaachen.de (N.G.); danielakroy@gmail.com (D.K.); hermann.wasmuth@luisenhospital.de (H.E.W.); pstrnad@ukaachen.de (P.S.); ctrautwein@ukaachen.de (C.T.); 2 Institute of Pathology, RWTH Aachen University, 52074 Aachen, Germany; sdjudjaj@ukaachen.de (S.D.); pboor@ukaachen.de (P.B.); 3 Institute of Molecular Pathobiochemistry, Experimental Gene Therapy and Clinical Chemistry, RWTH University Hospital Aachen, 52074 Aachen, Germany; rweiskirchen@ukaachen.de; 4 Rheumatology Section of the Department of Internal Medicine, Yale University School of Medicine, New Haven, CT 06520-8031, USA; richard.bucala@yale.edu; 5 Chair of Vascular Biology, Institute of Stroke and Dementia Research, LMU Klinikum, Lud-wig-Maximilian-University (LMU), 81377 Munich, Germany; juergen.bernhagen@med.uni-muenchen.de; 6 Munich Cluster for Systems Neurology, 81377 Munich, Germany

**Keywords:** MIF, NKT cells, NASH, liver fibrosis

## Abstract

Macrophage migration inhibitory factor (MIF) is a pleiotropic inflammatory cytokine with anti-fibrotic properties in toxic liver injury models and anti-steatotic functions in non-alcoholic fatty liver disease (NAFLD) attributed to the CD74/AMPK signaling pathway. As NAFLD progression is associated with fibrosis, we studied MIF function during NAFLD-associated liver fibrogenesis in mice and men by molecular, histological and immunological methods in vitro and in vivo. After NASH diet feeding, hepatic *Mif* expression was strongly induced, an effect which was absent in *Mif*^∆*hep*^ mice. In contrast to hepatotoxic fibrosis models, NASH diet-induced fibrogenesis was significantly abrogated in *Mif*^−/−^ and *Mif*^∆*hep*^ mice associated with a reduced accumulation of the pro-fibrotic type-I NKT cell subpopulation. In vitro, MIF skewed the differentiation of NKT cells towards the type-I subtype. In line with the murine results, expression of fibrosis markers strongly correlated with MIF, its receptors, and markers of NKT type-I cells in NASH patients. We conclude that MIF expression is induced during chronic metabolic injury in mice and men with hepatocytes representing the major source. In NAFLD progression, MIF contributes to liver fibrogenesis skewing NKT cell polarization toward a pro-fibrotic phenotype highlighting the complex, context-dependent role of MIF during chronic liver injury.

## 1. Introduction

Chronic liver diseases are a common health burden worldwide resulting in fibrosis and consecutive cirrhosis [1]. Chronic liver injuries thereby initiate an inflammatory response, which triggers the trans-differentiation of hepatic stellate cells (HSCs) from a quiescent vitamin A-storing phenotype into a highly activated state. Activated stellate cells produce an excess of extracellular matrix proteins leading to excessive scarring and fibrogenesis [2,3]. At the same time, the inflammatory response elicits the recruitment and accumulation of immune cell populations such as monocytes/macrophages, T cells, natural killer (NK) cells, and natural killer T (NKT) cells [4]. Cytokines and chemokines produced by liver resident cells including hepatocytes, activated stellate cells, biliary epithelial cells, and endothelial cells as well as infiltrating immune cells are key players in this process [5,6]. These processes finally shape the intrahepatic microenvironment in a highly dynamic manner [4].

Macrophage migration inhibitory factor (MIF) is a pleiotropic inflammatory cytokine with chemokine-like functions and serves as an upstream regulator of the innate immune response. Upon inflammatory and stress stimuli, MIF is secreted by several immune cells, as well as endothelial cells, thrombocytes, and selected parenchymal cells, e.g., adipocytes, keratinocytes, and fibroblasts [7,8]. Inflammatory effects of MIF include MAP kinase activation, immune cell recruitment, enhancement of macrophage survival, and inflammatory cytokine and chemokine production [7,9]. Distinct MIF signaling and target cell effects are mediated by high affinity interactions with the receptor CD74/invariant chain (Ii) or the chemokine receptors CXCR2 and CXCR4 [10,11]. Owing to its inflammatory spectrum of action, MIF is a pivotal mediator of inflammatory conditions and autoimmune diseases such as sepsis [12], rheumatoid arthritis [13,14], obesity [15], systemic lupus erythematosus [16], atherosclerosis [10,17], proliferative glomerulonephritis [18], autoimmune hepatitis [19], and alcohol-induced steatohepatitis [20].

In contrast, MIF is protective in a model of chronic injury-mediated renal fibrosis and a model of ischemia-reperfusion in the heart [21,22]. Likewise, we were recently able to show that MIF exhibits hepatoprotective effects in experimental models of hepatotoxin (carbon tetrachloride and thioacetamide)-induced chronic liver injury and fibrogenesis. The anti-fibrotic effect of MIF in these models was found to be mediated by the CD74/AMP-activated protein kinase (AMPK) signaling pathway in hepatic stellate cells and led to an attenuation of fibrogenic HSC activation [23]. Similarly, *Mif* gene-deficient (*Mif*^−/−^) mice exhibited an exacerbated phenotype in a chronic metabolic model of high-fat diet-triggered nonalcoholic hepatic steatosis. Protection from steatosis by MIF involved inhibition of hepatic fatty degeneration and was mediated through the CD74/AMPK axis in hepatocytes and a shift in hepatic macrophage polarization [24].

The hepatic microenvironment harbors various immune cells during homeostasis as well as injury including natural killer T (NKT) cells. Natural killer T (NKT) cells belong to the T lymphocyte class of immunocytes expressing T-cell receptors as well as surface markers that are indicative for natural killer (NK) cells [25]. In healthy livers, NKT cells are enriched in the sinusoidal area [26]. NKT cells recognize glycolipid antigens and are able to secrete either T helper cell 1 (Th1) or Th2 cytokines, when they are activated. Thus, NKT cells have the ability to influence the cytokine milieu within the liver toward a pro-fibrogenic response (via Th2 cytokines) or toward an anti-fibrogenic response (via Th1 cytokines), implicating a dichotomic, context-dependent role of NKT cells during chronic liver injury. Indeed, it has been shown that NKT cell accumulation is pro-fibrogenic in chronic viral hepatitis and primary biliary cirrhosis, whereas NKT cell accumulation acts anti-fibrogenic in a model of chronic carbon tetrachloride treatment [27,28]. These divergent functions might partly be attributed to a distinct predominance of a certain NKT subset. NKT cells have recently been classified into two major subsets, i.e., type I and type II NKT cells. Type I NKT cells can recognize cognate lipids and mostly exert inflammatory functions. The pro-fibrogenic responses of type I NKT cells have been linked to the activation of hepatic stellate cells via the Hedgehog (Hh) pathway, osteopontin expression and interferon-γ dependent recruitment of neutrophils and monocytes [29]. Additionally, type I NKT cells induce apoptosis of hepatocytes via FAS ligand (FASL), further aggravating liver injury [30]. In contrast, type II NKT cells protect the liver from injury and reverse the pro-inflammatory effect of type I NKT cells [30]. To distinguish between the two NKT subsets, the glycolipid α-GalCer can be used. α-GalCer strongly binds to CD1d on type I NKT cells specifically activating this subset [31].

Here, we studied the role of MIF during chronic metabolic liver injury and fibrosis in patients and mice and uncovered a previously unknown link between MIF and intrahepatic NKT subset polarization, shaping the fibrogenic response during the progression of NAFLD.

## 2. Materials and Methods

We confirm that all animal experiments and experimental protocols were performed and approved in accordance with the guidelines of the animal welfare review board (LANUV) of the federal government of North-Rhine Westfalia. All mouse strains and feedings were approved by the animal welfare review board (LANUV) of the federal government of North-Rhine Westfalia.

The human studies and methods we performed were approved by the local ethics committee of the University of Aachen. All experiments were conducted in accordance with the Declaration of Helsinki and the International Ethical Guidelines for Biomedical Research Involving Human Subjects. All participants provided a written informed consent. Liver tissue was collected from resected liver tissue of these patients between March 2014 and May 2017 at the Department of Internal Medicine III, University of Aachen, Germany.

### 2.1. Murine In Vivo Experiments and Determination of Hepatic Fibrosis

C57BL/6 *Mif*^−/−^ mice [32] were previously established in our laboratory [10]. Male C57BL/6J wild-type (8–10 weeks) mice were purchased from Charles River Laboratories and served as controls for the C57BL/6 *Mif*^−/−^ mice. For the generation of *Mif*^∆*hep*^ mice, the hepatocyte-specific Cre-recombinase (Alfp-cre) deleter line was crossed with *Mif^flox^*^/*flox*^ mice (>10 backcrosses) [33]. Alfp-cre negative *Mif^flox^*^/*flox*^ littermates served as controls for all *Mif*^∆*hep*^ mice experiments and were housed together during the experiments. All strains (*n* = 6–10 per group) were fed a methionine and choline-deficient diet (MCD, Ssniff Spezialdiäten GmbH, Soest, Germany) for eight weeks, representing a mouse model of diet-induced NASH. Liver fibrosis was histologically assessed by quantification of a Sirius red-positive area in 10 low-power fields (magnification: 200×) per slide using the software Image J/NIH. Intrahepatic levels of hydroxyproline were photometrically measured as described previously [34].

### 2.2. Hepatic Immune Cell Isolation and Flow Cytometry Analysis

Single-cell suspensions were isolated from freshly harvested murine livers by mechanical and enzymatic digestion as previously described [6]. For flow cytometry analysis, cell suspensions were stained with fluorochrome-conjugated antibodies for CD45, Ly6G, CD103, Ly6C, B220, NK1.1, Counting beads (BD Bioscience, Heidelberg, Germany), CD11b, F4/80, CD11c, MHCII, CD3, CD4, CD8, CD25, CD62L, FoxP3, viability dye (eBioscience, San Diego, CA, USA), α-GalCer (NIH) and analyzed using a LSR Fortessa flow cytometer system (BD Bioscience, Heidelberg, Germany). NKT cells were gated as singlets, viable, CD45+, CD3+ and NK1.1+ signals. For detailed gating strategy of all immune subsets see Supplementary Table S2. Data were analyzed using FlowJo software (Tree Star, Ashland, OR, USA).

### 2.3. Isolation and Cultivation of Murine NKT Cells

Murine NKT cells were isolated from the spleen of 10 weeks old *Mif*^−/−^ mice according to the protocol of a NK1.1 iNKT cell isolation kit (Miltenyi Biotec; Bergisch Gladbach; Germany) with magnetic beads mediated cell separation. After isolation, cells were resuspended in RPMI supplemented with 10% FCS, penicillin-streptomycin (100 U/mL), 15 mM Hepes, 0.1 mM non-essential amino acids, 1 mM sodium pyruvate and 50 µM 2-mercaptoethanol (Invitrogen, Carlsbad, CA, USA) and plated on plastic dishes.

Cells were stimulated with 50 ng/mL recombinant murine MIF prepared as described previously [35] or recombinant murine IL-12 (PeproTech, Rocky Hill, NJ, USA) for 24 h.

### 2.4. NAFLD Patient Cohort

Overall, 22 Caucasian patients with defined, distinct stages of NAFLD were included in the study cohort. Resection was performed for hepatocellular carcinoma, cholangiocellular carcinoma or metastasis to the liver of other tumor entities. Only liver tissue with maximal distance to the tumorous tissue was included in the analysis. Patients’ characteristics are depicted in Supplementary Table S1. Fibrosis was scored according to Kleiner et al. (0–4 score, [36]).

Total hepatic mRNA was isolated from liver tissue of included patients as previously described [37]. Gene expression analysis of liver fibrosis markers, NKT cell marker and MIF family members was performed via qRT-PCR using an ABI PRISM 7300 sequence detection system (Applied Biosystems, Foster City, CA, USA). For primer sequences see Supplementary Table S3.

### 2.5. Statistical Analysis

Data are represented as means ± SEM. Continuous variables were compared by two-sided T-test with Welch’s correction in case of unequal variances. Values of *p* < 0.05 were considered significant. The correlation studies were analyzed with Pearson correlation/linear regression between two variables. R-values indicated the goodness of fit. The *p*-value depicts on whether the correlation coefficient is significantly different from 0. *p*-values < 0.05 were considered significant. Statistical analyses were performed with GraphPad Prism 5.0 (GraphPad, San Diego, CA, USA).

## 3. Results

### 3.1. Intrahepatic Expression of MIF and Its Receptors Is Associated with NASH in Mice and Men

**Figure 1:**
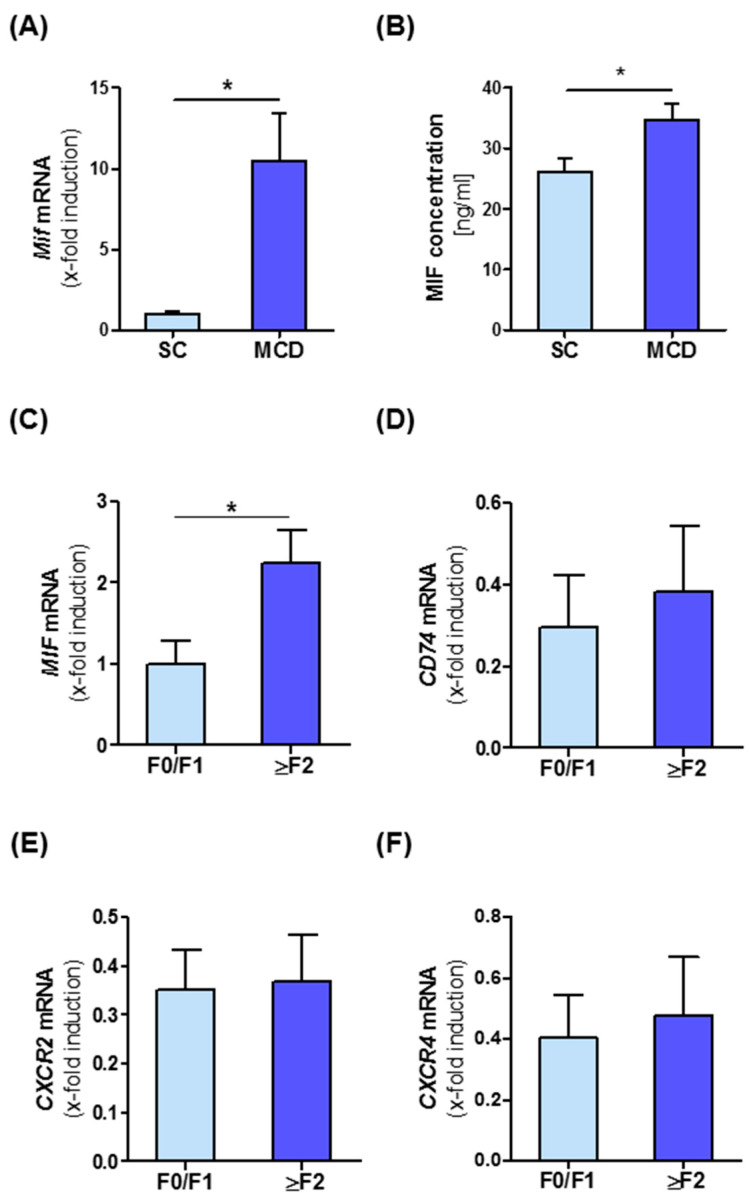
Intrahepatic migration inhibitory factor (MIF) expression is associated with progression of NASH in mice and human. (**A**) Expression of *Mif* mRNA in total liver tissue of wild-type (WT) mice which were fed with the methionine- and choline-deficient diet (MCD) diet for 8 weeks compared to standard chow-fed mice was measured by qRT-PCR (*n* = 6 per group). (**B**) Expression of MIF on protein levels was measured by ELISA between the two groups (*n* = 6 per group). (**C**–**F**) Expression of *MIF*, *CD74*, *CXCR2 and CXCR4* mRNA in different fibrosis grades in patients. Asterisks indicate statistical significance: * *p* < 0.05.

To get an insight into the potential role of MIF during NASH progression, we first analyzed intrahepatic MIF levels in a murine diet-induced model of NASH. MIF expression in whole liver tissue at both the mRNA and protein level was compared between C57BL/6J WT mice subjected to a methionine- and choline-deficient diet (MCD) for eight weeks and mice on standard chow (SC). Quantitative RT-PCR analysis revealed that *Mif* mRNA expression was substantially elevated (10-fold) after the MCD diet (Figure 1A). This notion was mirrored by a 1.3-fold increase in MIF protein in the livers of MCD diet-treated animals (Figure 1B). To verify if MIF induction during NASH progression is conserved across species, we analyzed intrahepatic *MIF* expression in a cohort of 22 patients comprising the broad spectrum of NASH from simple steatosis to advanced NASH-associated fibrosis. In NAFLD patients, *MIF* is up-regulated in higher fibrosis grades (2 and higher) compared to no or only mild fibrosis (grade 0 and 1) (Figure 1C). We also analyzed intrahepatic expression levels of the MIF receptors (*CD74*, *CXCR2* and *CXCR4*) in NASH patients, but could not detect a significant alteration, when stratifying patients according to fibrosis grade (Figure 1D–F). When correlating intrahepatic mRNA expression of the receptors with expression of surrogate markers for fibrogenesis in this cohort, we observed a positive for *CD74* (Figure S1B,C) and *CXCR2* (Figure S1D,E) with markers such as *ACTA2* (Figure S1B,D) and *TGF-β* (Figure S1C,E), while *MIF* expression positively correlated with intrahepatic expression of *COL1a1* (Figure S1A, for further correlation also see Supplementary Table S4).

### 3.2. Mif^−/−^ Mice Display Reduced Liver Fibrogenesis after Eight Weeks of MCD Diet

**Figure 2:**
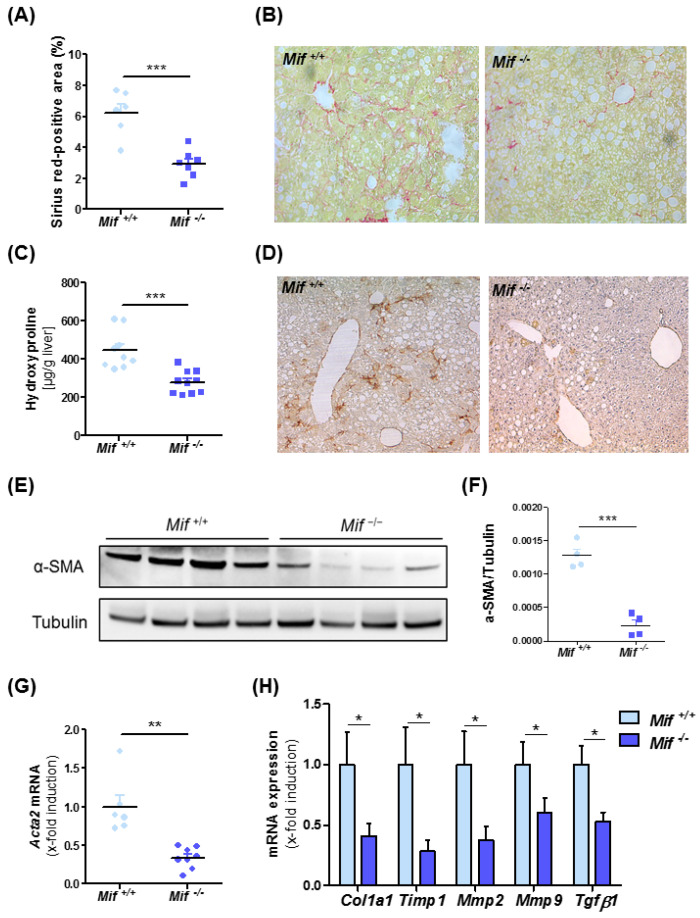
Decreased liver fibrosis progression in *Mif*-deficient mice in an experimental NASH model. (**A**) Quantification and (**B**) representative images of sirius red staining of WT and *Mif*^−/−^ mice after 8 weeks of MCD feeding. (**C**) Decreased fibrosis in *Mif*^−/−^ mice compared to WT (*n* = 10 per group) was confirmed by significantly decreased concentrations of hydroxyproline within the liver. (**D**) Immunhistochemical stainings for α-SMA in liver samples of WT and *Mif*^−/−^ mice after 8 weeks of MCD feeding. (**E**) Immunoblot analysis on whole liver tissue of WT and *Mif*^−/−^ mice after 8 weeks of MCD feeding using antibodies against α-SMA, tubulin served as loading control. (**F**) Relative α-SMA protein level. (**G**) Relative *Acta2* mRNA expression in total liver tissue of WT and *Mif*^−/−^ mice after 8 weeks of MCD feeding (*n* = 10 per group) quantitated using qRT-PCR (fold induction normalized to control animals). (**H**) Expression patterns of fibrosis-related genes, e.g., *Col1a1*, *Timp1*, *Mmp2*, *Mmp9*, and *Tgf-β1*, were measured by qRT-PCR between *Mif^+^*^/*+*^ mice and *Mif*^−/−^ mice (*n* = 10 per group). Asterisks indicate statistical significance: * *p* < 0.05; ** *p* < 0.01; *** *p* < 0.001.

To address the functional implications of enhanced MIF expression in NASH-associated liver fibrogenesis, WT mice were compared with constitutive *Mif*-deficient mice (*Mif*^−/−^ mice) in the MCD diet-feeding NASH model. Unexpectedly, *Mif*^−/−^ mice displayed substantially less fibrogenesis than WT mice over 8 weeks on diet as evidenced by diminished Sirius red-positive areas (Figure 2A,B) and decreased intrahepatic contents of the collagen-specific amino acid hydroxyproline (Figure 2C). WT and *Mif*^−/−^ mice after 8 weeks of normal diet feeding displayed no spontaneous phenotype with regard to fibrosis (Figure S2).

### 3.3. Reduced Liver Fibrosis in Mif^−/−^ Mice Is Associated with Reduced Expression of Pro-Fibrotic Genes and a Reduction of Activated Hepatic Stellate Cells

We next assessed the activation state of HSCs after MCD diet-induced NASH in the *Mif*-deficient setting. Immunohistochemical staining for α-SMA, a characteristic marker for activated stellate cells, was markedly reduced in liver sections of *Mif*^−/−^ mice as compared to control mice (Figure 2D). This finding was confirmed by quantification of *Acta2*/α-SMA content (Figure 2E–G). We next compared the expression levels of prototypical fibrosis-related genes implicated in the deposition and turnover of extracellular matrix proteins (*Col1a1*, *Timp1*, *Mmp2*, *Mmp9* and *Tgf-β1*) in the livers of WT and *Mif*^−/−^ mice. Figure 2H shows that the expression of all genes was markedly decreased in *Mif*^−/−^ mice when compared to MCD-fed WT mice, confirming the ameliorated fibrotic phenotype observed in MCD-fed *Mif*^−/−^ mice.

### 3.4. Hepatocytes Are the Main Source of MIF within the Liver

**Figure 3:**
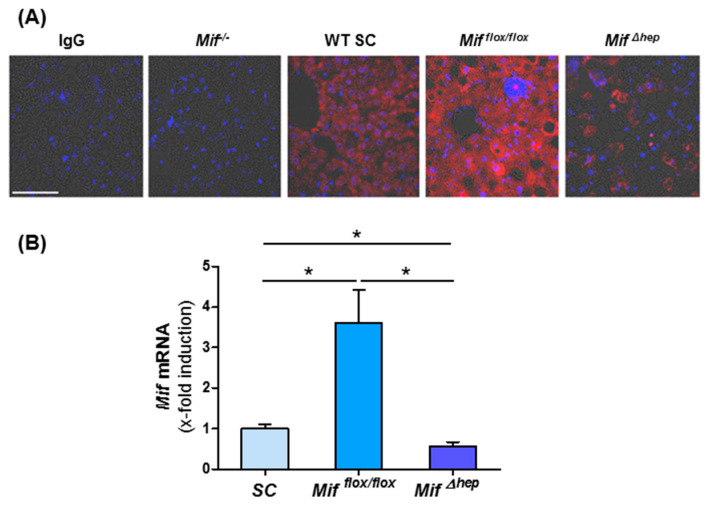
Hepatocytes are the main source of MIF in the liver. (**A**) Representative immunofluorescence stainings of WT mice after standard chow and after 8 weeks of MCD diet-induced NASH. IgG serves as antibody control and tissue from *Mif*^−/−^ and Mif^∆hep^ mice after 8 weeks of MCD diet serves as negative control (Scale bar 50 µm). (**B**) *Mif* mRNA expression in Alfp-cre negative *Mif^flox^*^/*flox*^ mice after MCD diet in contrast to standard chow fed WT mice and mRNA level of *Mif* in *Mif*^Δ*hep*^ mice of total liver lysates after 8 weeks of MCD diet compared to *Mif^flox^*^/*flox*^ mice were determined via qRT-PCR (*n* = 6 per group). Asterisks indicate statistical significance: * *p* < 0.05.

To define the main source of MIF in the liver during NASH progression, we stained liver sections from WT mice on standard chow and after eight weeks of MCD diet-induced NASH for MIF. Liver tissue of constitutively *Mif*-deficient mice and mice lacking *Mif* specifically in hepatocytes (*Mif*^∆*hep*^) fed 8 weeks of MCD diet served as controls. We found that strongly induced MIF protein expression in WT mice after MCD diet is mainly localized in hepatocytes (Figure 3A). To confirm the data from our immunofluorescence stainings, we performed qRT-PCR analysis. These data corroborated evidence on the induction of *Mif* during NASH, which was nearly completely abolished in the livers of mice specifically lacking *Mif* in hepatocytes (Figure 3B).

### 3.5. Specific Deletion of Mif in Hepatocytes Results in Reduced NASH-Mediated Liver Fibrogenesis Similar to Effects Observed in Constitutive Mif-Deficient Mice

**Figure 4:**
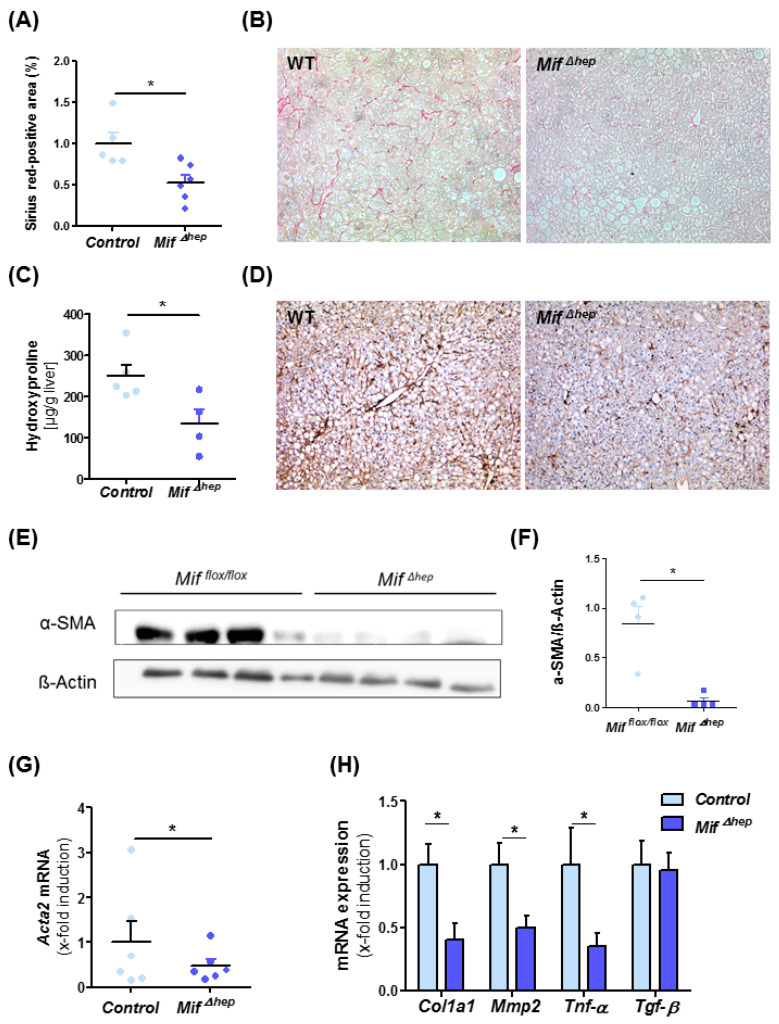
Decreased liver fibrosis progression in *Mif*^Δ*hep*^ mice after experimental NASH model. (**A**) Quantification and (**B**) representative Sirius red stainings of *Mif*^Δ*hep*^ and control littermates mice after 8 weeks of MCD feeding. Sirius-red stainings were quantitated using ImageJ software. (**C**) Biochemical measurement of amino acid hydroxyproline from snap-frozen liver tissue confirmed the decreased fibrosis level within the liver. (**D**) Representative immunohistochemical α-SMA stainings of *Mif*^Δ*hep*^ mice and control littermates after 8 weeks of MCD feeding and (**E**,**F**) Western blot analysis on whole liver tissue using antibodies against α-SMA, ß-Actin served as loading control. (**F**) Relative α-SMA protein level. (**G**) Hepatic stellate cells (HSC) activation is determined by mRNA expression level via qRT-PCR (*n* = 6 per group). (**H**) Expression patterns of fibrosis-related genes, e.g., *Col1a1*, *Mmp2*, *Tnf-α*, and *Tgf-β* were measured by qRT-PCR between *Mif*^Δ*hep*^ mice and control littermates (*n* = 6 per group). Asterisks indicate statistical significance: * *p* < 0.05.

After identifying hepatocytes as a major source of MIF during MCD-induced NASH in mice, we aimed to assess if hepatocyte-derived MIF is also a major functional contributor to liver fibrosis progression. For this, we treated *Mif*^∆*hep*^ mice and control littermates (*Alfp cre* negative) for eight weeks with MCD diet. *Mif*^∆*hep*^ mice exerted a similar phenotype as the mice with a global *Mif* deficiency with reduced levels of Sirius red-positive areas of stained liver sections (Figure 4A,B) and intrahepatic hydroxyproline levels (Figure 4C). In line with results obtained in *Mif*^−/−^ mice, activation of hepatic stellate cells was markedly ameliorated in the *Mif*^∆*hep*^ mice compared to control mice (Figure 4D–G, Figure S3). These findings were supported by altered gene expression of fibrosis-related genes such as *Col1a1*, *Mmp2* and *Tnf-α* (Figure 4H).

### 3.6. Hepatocyte-Specific Mif Deletion Leads to an Altered Pattern of Hepatic NKT Cell Polarization in MCD-Induced Liver Fibrogenesis

**Figure 5:**
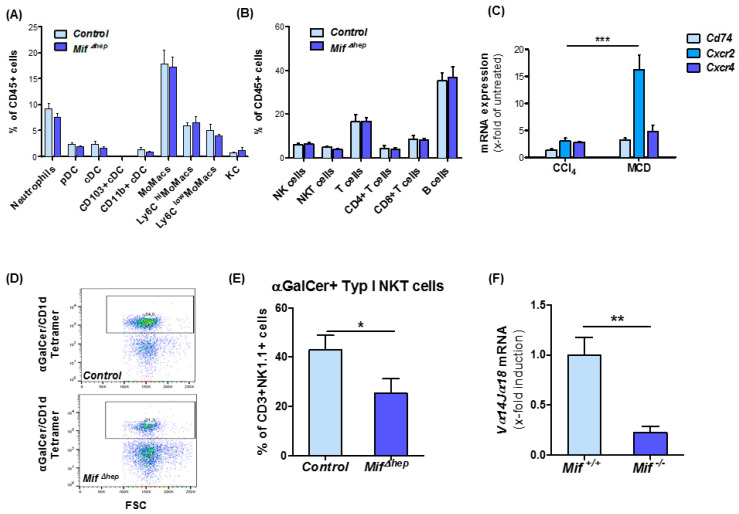
*Mif*^Δ*hep*^ mice show reduced type I natural killer T (NKT) cell infiltration. (**A**) Analysis of myeloid cells in the liver and (**B**) lymphoid populations in the liver after 8 weeks of MCD diet-induced NASH in *Mif*^Δ*hep*^ mice compared to control littermates in flow cytometry analysis. (**C**) MIF receptors analysis was performed by qRT-PCR after 8 weeks of MCD feeding compared to 6 weeks of CCl_4_ treatment from snap-frozen whole liver tissue. (**D**) Representative multicolor flow cytometry blots of type I NKT cell population. (**E**) Analysis of type I NKT cells in liver after 8 weeks MCD treatment in *Mif*^Δ*hep*^ mice compared to control littermates. Type I NKT cells were gated as viable cells, single cells, CD45+, CD3+, NK1.1+ and α-GalCer/CD1d Tetramer+ signals. (**F**) Expression pattern of type I NKT related gene *Vα14Jα18* was measured by qRT-PCR between *Mif*^Δ*hep*^ mice and control littermates. All analyses were performed with *n* = 6 mice per group. Asterisks indicate statistical significance: * *p* < 0.05; ** *p* < 0.01; *** *p* < 0.001.

Progression of liver fibrosis is paralleled by the recruitment of various immune cells and subsequent alterations of the intrahepatic immune cell repertoire contributing to fibrosis progression. Hence, we evaluated potential differences in the immune cell content in the liver of *Mif*^∆*hep*^ mice and controls after MCD diet-induced NASH. Performing a comprehensive analysis of the intrahepatic immune cell repertoire by flow cytometry analysis, we were unable to observe significant changes between the two groups of mice after MCD diet (Figure 5A,B), which is in line with our previously published data on immune cell alterations in global *Mif*-deficient mice in different models of toxin-induced liver injury [23].

However, interestingly, when we compared the injury-dependent induction of the expression of the MIF receptors *Cd74*, *Cxcr2*, and *Cxcr4* during toxin-induced liver injury and MCD-induced NASH, we identified a more than 15-fold induction of *Cxcr2* selectively in the NASH model, whereas *Cxcr4* and *Cd74* were only slightly induced in the liver in both models (Figure 5C). Furthermore, in isolated NKT cells from *Mif*^−/−^ mice, the expression level of *Cxcr2* and *Cxcr4* is increased after 24 h of stimulation with recombinant, murine MIF (Figure S4A,B). Of note, CXCR2 has been shown to mediate NKT cell accumulation by MIF to the tissue in a model of skin inflammation [38]. With regard to these findings and the established functional role of NKT subsets in liver fibrosis, we proceeded to further characterize the NKT subset repertoire in our NASH model. Using multicolor flow cytometry analysis, we detected a significantly decreased accumulation of the type I NKT cell subset in the *Mif*^∆*hep*^ mice compared to control mice (Figure 5D,E). This observation could also be extended to global *Mif*^−/−^ mice by assessing mRNA expression levels of type I NKT specific marker *Vα14Jα18* in *Mif*^−/−^ mice and corresponding controls after MCD diet feeding (Figure 5F). In line with these data, the functionality of type I NKT cells might be impaired in *Mif*^Δ*hep*^ mice, which is implicated by diminished mRNA expression levels of *T-bet*, *Cxcr3* and *Il-17* (Figure S5A–C).

### 3.7. Intrahepatic Expression of Fibrosis-Related Genes Is Linked to NKT Cell Subset Marker Expression in Human NASH

**Figure 6:**
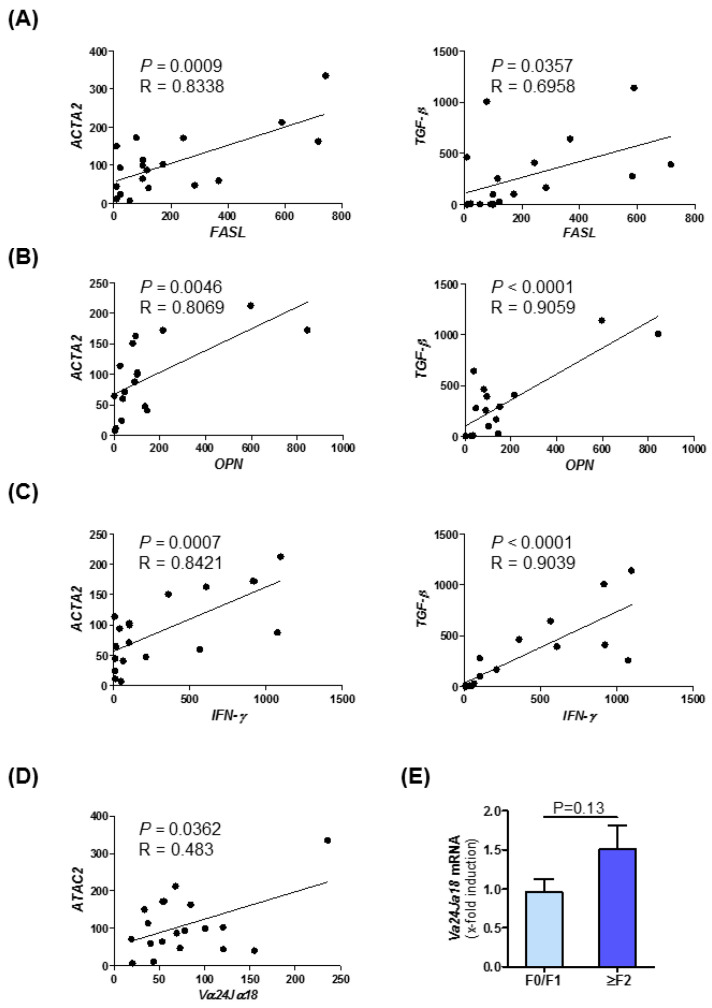
Expression of NKT cell marker correlates with fibrosis markers of patients with non-alcoholic steatohepatitis. Correlation analyses of mRNA expression level in NASH patient samples were performed by qRT-PCR analysis. There were 22 human samples from liver resections with different stages of steatosis and fibrosis analyzed. (**A**) NKT-associated *FASL* expression was correlated with *ACTA2* (left panel) and TGF-β (right panel) expression. (**B**) Fibrosis-related expression of *ACTA2* (left panel) and *TGF-β* (right panel) were correlated with *OPN* expression. (**C**) Correlation analysis between *ACTA2* (left panel) and *TGF-β* (right panel) with inflammatory marker *IFN-γ* (**D**) Expression of *ACTA2* were correlated with the type I NKT related gene *Vα24Jα18* via qRT-PCR. (**E**) Expression pattern of *Vα24Jα18* was measured by qRT-PCR in different fibrosis grades in patients.

Furthermore, in samples of NASH patients, we could observe strong correlations between the expression of NKT cell markers and fibrosis markers. In this correlation analysis, we observed a strong positive association between the expression of NKT type I markers like *FASL* (Figure 6A), *OPN* (Figure 6B), and *Interferon-γ* (Figure 6C) and fibrosis and inflammatory markers such as *ACTA2* and *TGF-β* [39]. These data and the correlations determined by Pearson correlation/linear regression analysis displayed in Figure 1C–F indicate a conserved association of MIF, fibrosis progression and NKT cell in human NASH. A summary of correlation analysis of intrahepatic mRNA expression level of *MIF*, its receptors *CXCR2*/*CXCR4*/*CD74*, NKT cell marker and fibrosis-associated genes in NAFLD patients samples are shown in Table S4. Moreover, we have assessed the mRNA expression of the NKT type I specific marker *Vα14Jα18*, the human homolog of *Vα24Jα18*, in the patient cohort. Figure 6D depicts the expression of this marker stratified by *ACTA2* expression. Furthermore, we could evidence a trend towards higher levels in the ≥F2 group (Figure 6E).

### 3.8. MIF Directly Modulates NKT Cell Polarization

**Figure 7:**
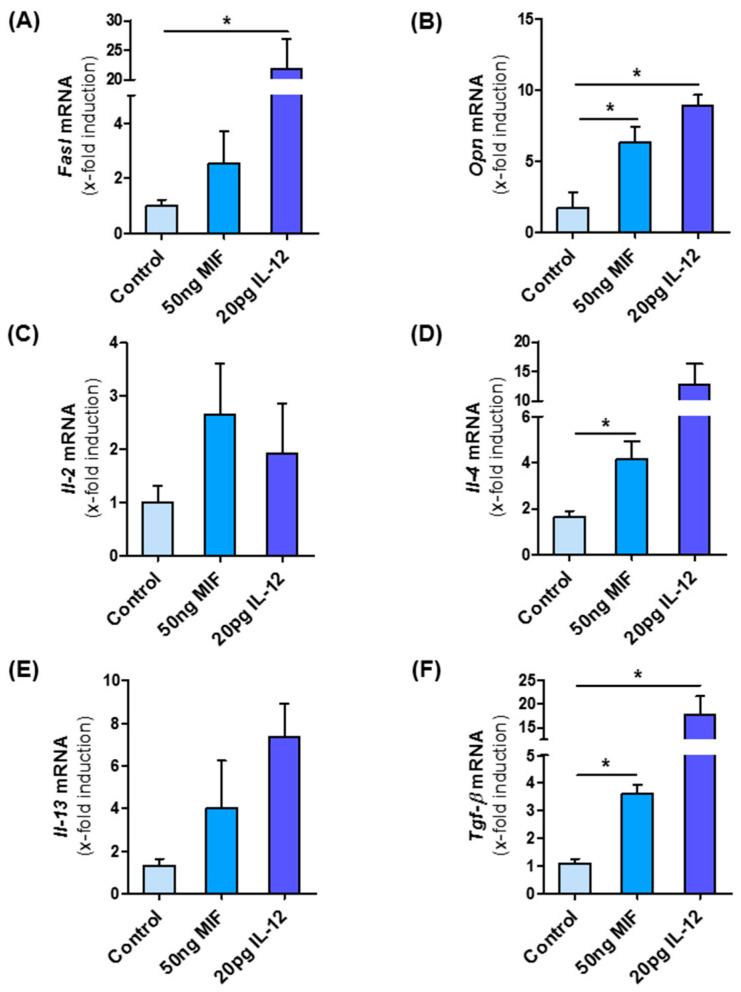
NKT cells are skewed to pro-fibrotic type I subpopulation by MIF. NKT cells were isolated from the spleen from untreated, 10 weeks old, male *Mif*^−/−^ mice. NKT cells were isolated via one negative and one positive selection with the MACS separator. Isolated cells were stimulated in vitro with 50 ng/mL of recombinant, murine MIF and as positive control with 20 pg/mL IL-12 for 24 h. After stimulation, mRNA analysis was performed to determine the expression levels of type I NKT cell marker (**A**) *Fasl* and (**B**) *Opn*. The expression levels of (**C**–**E**) Interleukins and *Tgf-β* (**F**), which is expressed by type I NKT cells, are also determined by qRT-PCR after MIF stimulation. Experiments were performed two times with four technical replicates. Asterisks indicate statistical significance: * *p* < 0.05.

As *Mif*^∆*hep*^ mice show decreased type I NKT cells, we finally asked whether MIF might directly influence NKT cell polarization and thereby modulate the intrahepatic microenvironment during NASH. To address this hypothesis, we isolated NKT cells from *Mif*^−/−^ mice to exclude bias from intrinsic MIF expression by the cells and incubated them with recombinant murine MIF for 24 h. As a positive control, we stimulated the NKT cells with recombinant murine IL-12, a known activator of type I differentiation. After MIF stimulation of NKT cells, we observed an increase in mRNA expression of *Fasl* and *Opn* (Figure 7A,B); two factors linked to the pro-fibrotic function of type I NKT cells. Furthermore, specific interleukins, e.g., *Il-2*, *Il-4* and *Il-13*, which are associated with type I NKT subset were up-regulated in the same manner as after the stimulation with IL-12 (Figure 7C–E) as well as the pro-fibrotic cytokine and HSC stimulator *Tgf-β* (Figure 7F). Of note, TGF-β secretion is also a hallmark of type I NKT differentiation [39].

**Figure 8:**
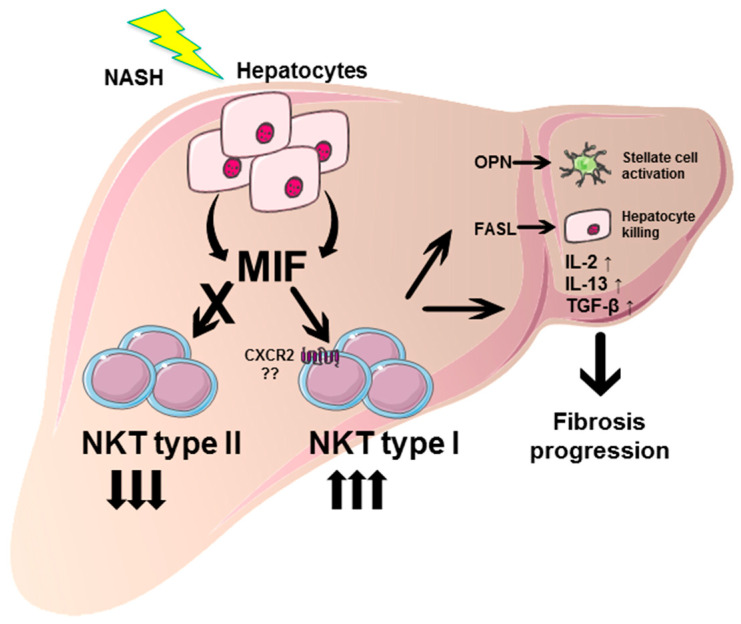
Graphical abstract summarizing the pro-fibrotic role of MIF in NASH and proposed underlying mechanisms. During NASH pathogenesis, MIF expression is strongly induced in hepatocytes. These increased intrahepatic MIF levels promote the differentiation of type I intrahepatic NKT cells, while differentiation towards type II NKT subpopulation is inhibited. Type I NKT cells exert pro-fibrotic properties, e.g., by secretion of OPN, activating hepatic stellate cells and consecutive collagen synthesis. Moreover, type I NKT cells perpetuate liver damage by FASL-mediated direct killing of hepatocytes, and orchestrate a pro-inflammatory and pro-fibrotic environment by expression of cytokines, e.g., IL-2, IL-13 and TGF-β resulting in progression of hepatic fibrosis in NASH. This figure was created using Servier Medical Art templates, which are licensed under a Creative Commons Attribution 3.0 Unported License; https://smart.servier.com.

Taken together, these data suggest that the pro-fibrotic effect of the cytokine MIF in NASH-induced fibrosis progression may—at least in part—be due to a direct effect on NKT cell polarization and perpetuation of the fibrogenic response (Figure 8).

## 4. Discussion

Intrahepatic expression of MIF was found to be strongly induced in an experimental model of NASH in mice and correlates with disease severity in patients with NAFLD. These results implicate a functional, conserved role of MIF in the progression of NAFLD. In contrast to the hepatoprotective role that this cytokine exhibits in hepatotoxin-induced liver fibrosis and high fat diet-induced fatty liver degeneration [23,24], the current study unexpectedly demonstrated a clear-cut pro-fibrogenic effect of MIF in the MCD model of NASH. The ameliorated fibrosis phenotype could also be observed in mice selectively lacking MIF in hepatocytes identifying the hepatocyte as the main, functionally relevant source of MIF during NASH. Our study thereby complements earlier in vitro studies from Marin et al. as well as bone marrow chimera experiments in a model of ethanol-induced liver injury identifying liver resident cells as a pivotal source of MIF in the liver [40]. Interestingly, while the reduction of fibrogenesis in globally *Mif*-deficient mice was quite accurately replicated in *Mif*^Δ*hep*^ mice after MCD diet-induced NASH, intrahepatic mRNA expression levels of the potent HSC-activating cytokine *Tgf-β* were only decreased in global *Mif*^−/−^ mice. Hence, MIF expression by non-hepatocytic sources seems to be sufficient to account for the increase of *Tgf-β* in the mouse model of MCD diet-induced NASH as compared to normal chow feed mice without affecting liver fibrogenesis per se to the same extent. These findings might implicate different thresholds for MIF-mediated effects in the liver or could be a result of local niche-specific effects.

In previous studies, we could show that MIF exerts direct inhibitory effects on HSC activation via the CD74/AMP kinase signaling pathway and that this function is a pivotal driver of its hepatoprotective effects in the hepatotoxin-induced fibrosis models [23]. Moreover, activation of the CD74/AMP kinase signaling in hepatocytes by MIF ameliorates high fat diet-induced fatty liver degeneration [24]. In addition to signaling via CD74, MIF also mediates effects via the chemokine receptors CXCR2 and CXCR4. Of note, when we compared the intrahepatic MIF receptor repertoire between the hepatotoxin- and MCD diet-induced injury models, we identified marked differences between the models. While the induction of the receptors CD74 and CXCR4 is comparable between the models, there is a marked up-regulation of chemokine receptor CXCR2 in MCD-treated mice compared to the CCl_4_-induced liver fibrosis model. CXCR2 mediates the recruitment of various leukocytes, e.g., granulocytes, NK cells, T cells and monocytes to the tissue able to modulate the fibrogenic response in non-alcoholic steatohepatitis [4]. We therefore analyzed the infiltration of various leukocyte subtypes into the liver. Unlike for a shifting in NKT subsets however, we observed no differences in the overall intrahepatic immune cell repertoire after MCD-induced NASH.

In MCD induced NASH, NKT depletion led to attenuated liver fibrosis compared to wild-type mice [29]. In contrast, in an acute model of acetaminophen-induced liver injury, NKT-deficient mice showed an increased susceptibility for liver injury [41]. However, both studies did not analyze the prevalence of NKT subsets in the specific model, which might substantially alter the outcome of NKT depletion strategies in these settings as NKT subsets convey opposing roles during liver injury [42]. In our study using the MCD diet-based NASH model, *Mif* gene deficiency led to strongly reduced levels of intrahepatic type I NKT cells as evidenced by flow cytometry analysis and lower expression of type I specific *Vα14Jα18*, indicating that MIF skews NKT cells polarization toward the more pro-inflammatory and pro-fibrotic type I phenotype. These findings were accompanied by reduced levels of *T-bet*, *Cxcr3* and *Il-17*, which might further implicate a decreased functionality of type I NKT cells [43], although these markers are not specific to type I NKT cells. Furthermore, in a translational correlation study, we identified a strong correlation between type I NKT-related gene expression and fibrosis markers in NASH patients, implicating that the regulatory mechanisms of MIF during NASH-induced fibrosis evidenced in our murine model are also present and relevant in human NASH.

In order to determine if MIF has a direct effect on NKT subset polarization, we stimulated primary NKT cells with recombinant murine MIF. mRNA expression analysis in these cells demonstrated a marked increase in the expression of genes that are associated with the type I NKT cells phenotype, which was similar to that induced by IL-12, an established inducer of type 1 NKT polarization.

Taken together, our findings demonstrated a pro-fibrotic effect of MIF in the model of MCD diet-induced NASH. Moreover, MIF was found to induce hepatic type I NKT cell skewing. However, if these two effects were directly and causally linked further experimental proof such as restoration of NKT subset polarization in *Mif*-deficient mice would be needed. Our results highlight the complex, in part even dichotomic role of MIF during chronic liver disease, which is of specific importance when considering MIF as a target of therapeutic interventions in patients with chronic liver disease. To this end, it is especially intriguing to note that during NAFLD, MIF protects from high fat diet as well as MCD diet-induced steatosis [24], but exacerbates liver fibrogenesis that ensues MCD-related NASH. Therefore, even within the same injury system, the effects of MIF seem to be context-dependent. This is in line with earlier observations in experimental models of ethanol-induced liver injury. While MIF exacerbates liver injury during chronic ethanol feeding [20,40], it mediates protective effects following chronic-binge ethanol feeding [44].

So what might mediate the overall impact of MIF in the various settings then? One possible explanation might be a distinct predominance of MIF-mediated immune cell recruitment/polarization versus its direct effects on liver resident cells (e.g., steatotic hepatocytes, HSC) inherent in the specific settings. Based on our observation, the specific intrahepatic MIF receptor expression pattern at a given setting might be a key determinant of MIF-mediated outcome in the different disease models. However, this will require further investigations and will be a prerequisite to guide MIF-directed therapeutic interventions. Additional studies are needed to determine efficient, valid, and stable parameters to predict the outcome of MIF targeting strategies (agonistic or antagonistic) for treatment of individual patients with chronic liver injury of distinct etiology and stages in clinical practice.

## Data Availability

No new data outside those presented in this study were created or analyzed. Data sharing is not applicable to this article.

## Supplementary Materials

The following are available online at https://www.mdpi.com/2073-4409/10/2/252/s1, Figure S1: Human correlation studies in a patient cohort comprising the broad spectrum of fibrosis-associated NASH, Figure S2: Sirius-Red staining of liver tissue from normal diet feed WT and *Mif*^−^^/−^ mice, Figure S3: Quantification of a-SMA stainings from *Mif*^Δ*hep*^, Figure S4: Expression patterns of MIF receptors in vitro, Figure S5: Expression patterns after eight weeks of MCD diet induced NASH. Table S1: Clinical parameters of all patients (*n* = 22) at admission, Table S2: Gating strategy, Table S3: Murine Primer used in this publication. Table S4: Summary of correlation analysis of intrahepatic mRNA expression level of MIF, its receptors *CXCR2/CXCR4/CD74*, NKT cell marker and fibrosis-associated genes in NAFLD patients’ samples as assessed by qRT-PCR analysis. 22 human samples with different stages of steatosis and fibrosis were analyzed.

## Abbreviations

MIF	Macrophage migration inhibitory factor
NAFLD	Non-alcoholic fatty liver disease
HSC	Hepatic stellate cells
NK cells	Natural killer cells
NKT cells	Natural killer T cells
AMPK	AMP-activated protein kinase
CCl_4_	Carbon tetrachloride
Hh	Hedgehog
FASL	FAS ligand
pDCs	Plasmacytoid DCs
MCD	Methionine- and choline-deficient
NASH	Non-alcoholic steatohepatitis
α-GalCer	α-Galactosylceramide

## References

[1] Tsochatzis E.A., Bosch J., Burroughs A.K. (2014). Liver cirrhosis. Lancet.

[2] Iredale J.P. (2007). Models of liver fibrosis: Exploring the dynamic nature of inflammation and repair in a solid organ. J. Clin. Investig..

[3] Tsuchida T., Friedman S.L. (2017). Mechanisms of hepatic stellate cell activation. Nat. Rev. Gastroenterol. Hepatol..

[4] Heymann F., Tacke F. (2016). Immunology in the liver-from homeostasis to disease. Nat. Rev. Gastroenterol. Hepatol..

[5] Marra F., Tacke F. (2014). Roles for chemokines in liver disease. Gastroenterology.

[6] Berres M.L., Koenen R.R., Rueland A., Zaldivar M.M., Heinrichs D., Sahin H., Schmitz P., Streetz K.L., Berg T., Gassler N. (2010). Antagonism of the chemokine Ccl5 ameliorates experimental liver fibrosis in mice. J. Clin. Investig..

[7] Calandra T., Roger T. (2003). Macrophage migration inhibitory factor: A regulator of innate immunity. Nat. Rev. Immunol..

[8] Schober A., Bernhagen J., Weber C. (2008). Chemokine-like functions of MIF in atherosclerosis. J. Mol. Med..

[9] Tillmann S., Bernhagen J., Noels H. (2013). Arrest Functions of the MIF Ligand/Receptor Axes in Atherogenesis. Front. Immunol..

[10] Bernhagen J., Krohn R., Lue H., Gregory J.L., Zernecke A., Koenen R.R., Dewor M., Georgiev I., Schober A., Leng L. (2007). MIF is a noncognate ligand of CXC chemokine receptors in inflammatory and atherogenic cell recruitment. Nat. Med..

[11] Leng L., Metz C.N., Fang Y., Xu J., Donnelly S., Baugh J., Delohery T., Chen Y., Mitchell R.A., Bucala R. (2003). MIF signal transduction initiated by binding to CD74. J. Exp. Med..

[12] Bernhagen J., Calandra T., Mitchell R.A., Martin S.B., Tracey K.J., Voelter W., Manogue K.R., Cerami A., Bucala R. (1993). MIF is a pituitary-derived cytokine that potentiates lethal endotoxaemia. Nature.

[13] Baugh J.A., Chitnis S., Donnelly S.C., Monteiro J., Lin X., Plant B.J., Wolfe F., Gregersen P.K., Bucala R. (2002). A functional promoter polymorphism in the macrophage migration inhibitory factor (MIF) gene associated with disease severity in rheumatoid arthritis. Genes Immun..

[14] Morand E.F., Leech M., Bernhagen J. (2006). MIF: A new cytokine link between rheumatoid arthritis and atherosclerosis. Nat. Rev. Drug Discov..

[15] Finucane O.M., Reynolds C.M., McGillicuddy F.C., Harford K.A., Morrison M., Baugh J., Roche H.M. (2014). Macrophage migration inhibitory factor deficiency ameliorates high-fat diet induced insulin resistance in mice with reduced adipose inflammation and hepatic steatosis. PLoS ONE.

[16] Sreih A., Ezzeddine R., Leng L., LaChance A., Yu G., Mizue Y., Subrahmanyan L., Pons-Estel B.A., Abelson A.K., Gunnarsson I. (2011). Dual effect of the macrophage migration inhibitory factor gene on the development and severity of human systemic lupus erythematosus. Arthritis Rheum..

[17] Zernecke A., Bernhagen J., Weber C. (2008). Macrophage migration inhibitory factor in cardiovascular disease. Circulation.

[18] Djudjaj S., Lue H., Rong S., Papasotiriou M., Klinkhammer B.M., Zok S., Klaener O., Braun G.S., Lindenmeyer M.T., Cohen C.D. (2016). Macrophage Migration Inhibitory Factor Mediates Proliferative GN via CD74. J. Am. Soc. Nephrol..

[19] Assis D.N., Leng L., Du X., Zhang C.K., Grieb G., Merk M., Garcia A.B., McCrann C., Chapiro J., Meinhardt A. (2014). The role of macrophage migration inhibitory factor in autoimmune liver disease. Hepatology.

[20] Barnes M.A., McMullen M.R., Roychowdhury S., Pisano S.G., Liu X., Stavitsky A.B., Bucala R., Nagy L.E. (2013). Macrophage migration inhibitory factor contributes to ethanol-induced liver injury by mediating cell injury, steatohepatitis, and steatosis. Hepatology.

[21] Djudjaj S., Martin I.V., Buhl E.M., Nothofer N.J., Leng L., Piecychna M., Floege J., Bernhagen J., Bucala R., Boor P. (2017). Macrophage Migration Inhibitory Factor Limits Renal Inflammation and Fibrosis by Counteracting Tubular Cell Cycle Arrest. J. Am. Soc. Nephrol..

[22] Miller E.J., Li J., Leng L., McDonald C., Atsumi T., Bucala R., Young L.H. (2008). Macrophage migration inhibitory factor stimulates AMP-activated protein kinase in the ischaemic heart. Nature.

[23] Heinrichs D., Knauel M., Offermanns C., Berres M.L., Nellen A., Leng L., Schmitz P., Bucala R., Trautwein C., Weber C. (2011). Macrophage migration inhibitory factor (MIF) exerts antifibrotic effects in experimental liver fibrosis via CD74. Proc. Natl. Acad. Sci. USA.

[24] Heinrichs D., Berres M.L., Coeuru M., Knauel M., Nellen A., Fischer P., Philippeit C., Bucala R., Trautwein C., Wasmuth H.E. (2014). Protective role of macrophage migration inhibitory factor in nonalcoholic steatohepatitis. FASEB J..

[25] Tajiri K., Shimizu Y. (2012). Role of NKT Cells in the Pathogenesis of NAFLD. Int. J. Hepatol..

[26] Syn W.K., Oo Y.H., Pereira T.A., Karaca G.F., Jung Y., Omenetti A., Witek R.P., Choi S.S., Guy C.D., Fearing C.M. (2010). Accumulation of natural killer T cells in progressive nonalcoholic fatty liver disease. Hepatology.

[27] Park O., Jeong W.I., Wang L., Wang H., Lian Z.X., Gershwin M.E., Gao B. (2009). Diverse roles of invariant natural killer T cells in liver injury and fibrosis induced by carbon tetrachloride. Hepatology.

[28] Durante-Mangoni E., Wang R., Shaulov A., He Q., Nasser I., Afdhal N., Koziel M.J., Exley M.A. (2004). Hepatic CD1d expression in hepatitis C virus infection and recognition by resident proinflammatory CD1d-reactive T cells. J. Immunol..

[29] Syn W.K., Agboola K.M., Swiderska M., Michelotti G.A., Liaskou E., Pang H., Xie G., Philips G., Chan I.S., Karaca G.F. (2012). NKT-associated hedgehog and osteopontin drive fibrogenesis in non-alcoholic fatty liver disease. Gut.

[30] Kumar V. (2013). NKT-cell subsets: Promoters and protectors in inflammatory liver disease. J. Hepatol..

[31] Liao C.M., Zimmer M.I., Wang C.R. (2013). The functions of type I and type II natural killer T cells in inflammatory bowel diseases. Inflamm. Bowel Dis..

[32] Fingerle-Rowson G., Petrenko O., Metz C.N., Forsthuber T.G., Mitchell R., Huss R., Moll U., Muller W., Bucala R. (2003). The p53-dependent effects of macrophage migration inhibitory factor revealed by gene targeting. Proc. Natl. Acad. Sci. USA.

[33] Brocks T., Fedorchenko O., Schliermann N., Stein A., Moll U.M., Seegobin S., Dewor M., Hallek M., Marquardt Y., Fietkau K. (2017). Macrophage migration inhibitory factor protects from nonmelanoma epidermal tumors by regulating the number of antigen-presenting cells in skin. FASEB J..

[34] Hillebrandt S., Wasmuth H.E., Weiskirchen R., Hellerbrand C., Keppeler H., Werth A., Schirin-Sokhan R., Wilkens G., Geier A., Lorenzen J. (2005). Complement factor 5 is a quantitative trait gene that modifies liver fibrogenesis in mice and humans. Nat. Genet..

[35] Bernhagen J., Mitchell R.A., Calandra T., Voelter W., Cerami A., Bucala R. (1994). Purification, bioactivity, and secondary structure analysis of mouse and human macrophage migration inhibitory factor (MIF). Biochemistry.

[36] Kleiner D.E., Brunt E.M., Van Natta M., Behling C., Contos M.J., Cummings O.W., Ferrell L.D., Liu Y.C., Torbenson M.S., Unalp-Arida A. (2005). Design and validation of a histological scoring system for nonalcoholic fatty liver disease. Hepatology.

[37] Nellen A., Heinrichs D., Berres M.L., Sahin H., Schmitz P., Proudfoot A.E., Trautwein C., Wasmuth H.E. (2012). Interference with oligomerization and glycosaminoglycan binding of the chemokine CCL5 improves experimental liver injury. PLoS ONE.

[38] Hsieh C.Y., Chen C.L., Lin Y.S., Yeh T.M., Tsai T.T., Hong M.Y., Lin C.F. (2014). Macrophage migration inhibitory factor triggers chemotaxis of CD74+CXCR2+ NKT cells in chemically induced IFN-gamma-mediated skin inflammation. J. Immunol..

[39] Doisne J.M., Bartholin L., Yan K.P., Garcia C.N., Duarte N., Le Luduec J.B., Vincent D., Cyprian F., Horvat B., Martel S. (2009). iNKT cell development is orchestrated by different branches of TGF-beta signaling. J. Exp. Med..

[40] Marin V., Poulsen K., Odena G., McMullen M.R., Altamirano J., Sancho-Bru P., Tiribelli C., Caballeria J., Rosso N., Bataller R. (2017). Hepatocyte-derived macrophage migration inhibitory factor mediates alcohol-induced liver injury in mice and patients. J. Hepatol..

[41] Martin-Murphy B.V., Kominsky D.J., Orlicky D.J., Donohue T.M., Ju C. (2013). Increased susceptibility of natural killer T-cell-deficient mice to acetaminophen-induced liver injury. Hepatology.

[42] Lee K.C., Chen P., Maricic I., Inamine T., Hu J., Gong S., Sun J.C., Dasgupta S., Lin H.C., Lin Y.T. (2019). Intestinal iNKT cells migrate to liver and contribute to hepatocyte apoptosis during alcoholic liver disease. Am. J. Physiol. Gastrointest. Liver Physiol..

[43] Maricic I., Marrero I., Eguchi A., Nakamura R., Johnson C.D., Dasgupta S., Hernandez C.D., Nguyen P.S., Swafford A.D., Knight R. (2018). Differential Activation of Hepatic Invariant NKT Cell Subsets Plays a Key Role in Progression of Nonalcoholic Steatohepatitis. J. Immunol..

[44] Poulsen K.L., McMullen M.R., Huang E., Kibler C.D., Sheehan M., Leng L., Bucala R., Nagy L.E. (2019). Novel Role of Macrophage Migration Inhibitory Factor in Upstream Control of the Unfolded Protein Response after Ethanol Feeding in Mice. Alcohol Clin. Exp. Res..

