# Supplementary material for: Unexpected Pro-Fibrotic Effect of MIF in Non-Alcoholic Steatohepatitis Is Linked to a Shift in NKT Cell Populations
Source: Cells. 2021 Jan 28;10(2):252. doi: 10.3390/cells10020252 (PMC7918903; doi:10.3390/cells10020252)
Supplement: Supplementary file 1 [file cells-10-00252-s001.zip › cells-1068813-supplementary-PUB/Supplementary Figures.pdf]

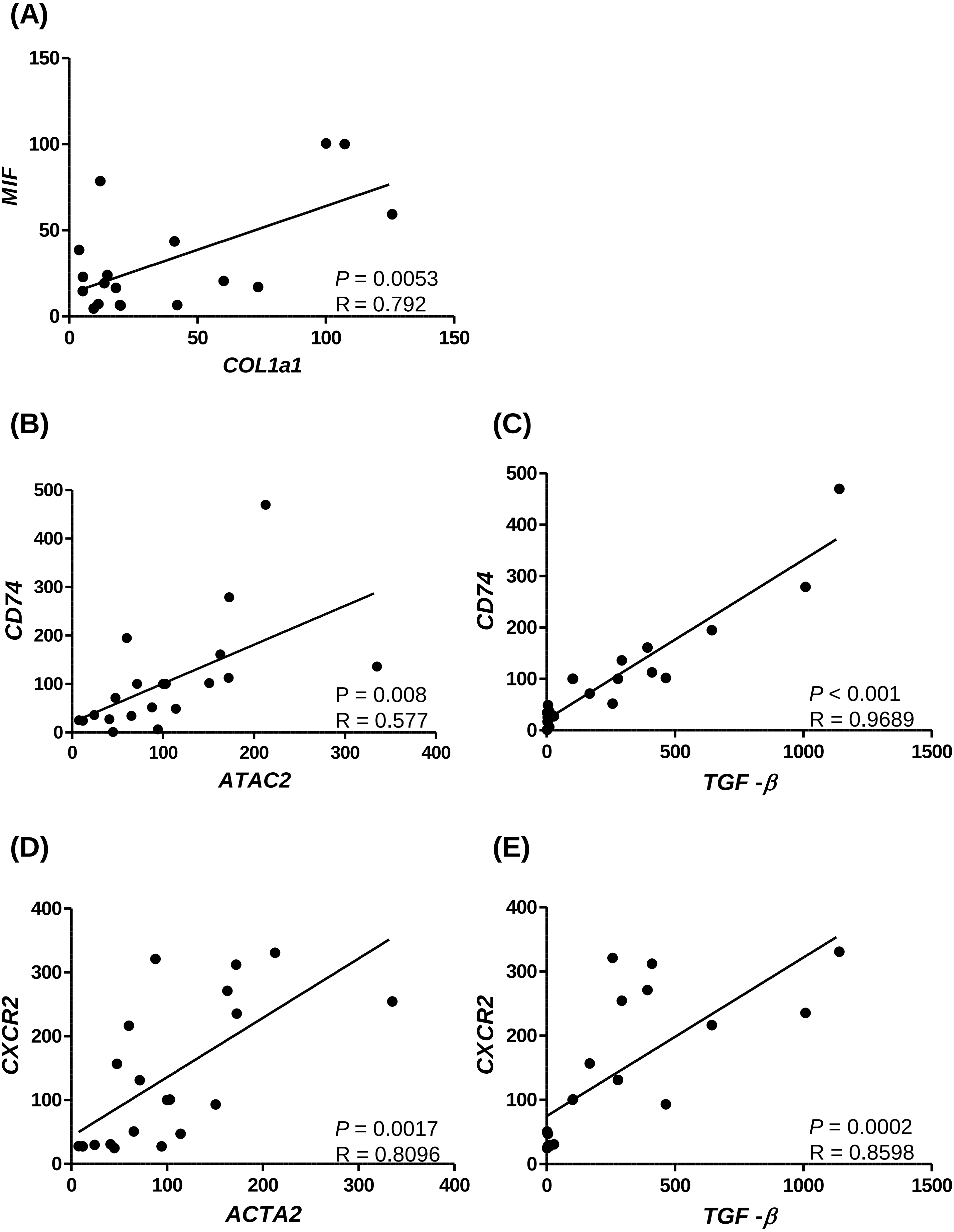

Supplementary Fig. 1. Human correlation studies in a patient cohort comprising the broad spectrum of fibrosis-associated NASH Correlation studies from 22 patients between *MIF* and *COL1a1* (A), *CD74* and *ACTA2* (B) *CD74* and *TGF-β* (C), *CXCR2* and *ACTA2* (D) and *CXCR2* and *TGF-β* (D) by Pearson correlation / linear regression analysis.

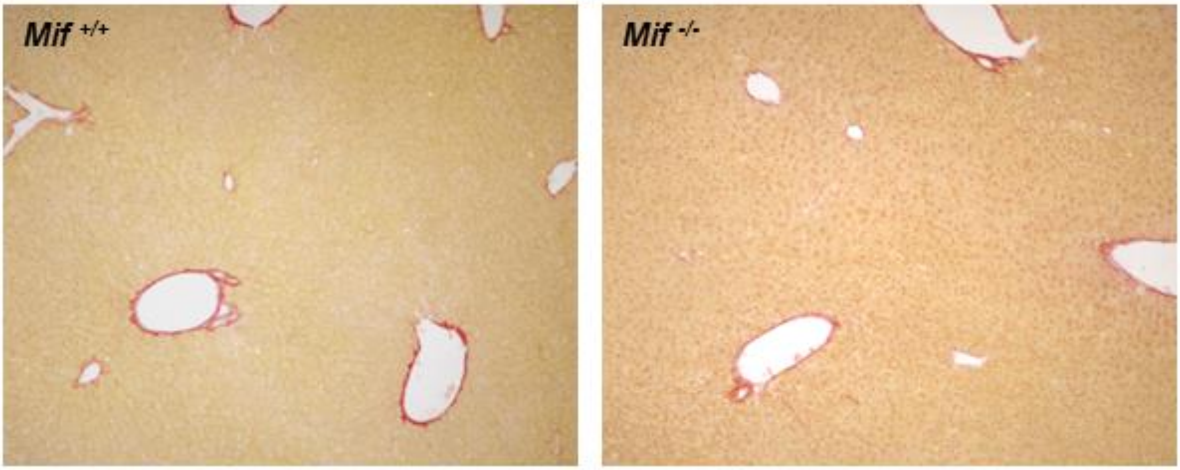

Supplementary Fig. 2. Sirius Red staining of liver tissue from normal diet feed WT and *Mif*$^{-/-}$ mice Representative Sirius-red stainings of WT and *Mif*$^{-/-}$ mice after 8 weeks of normal diet feeding.

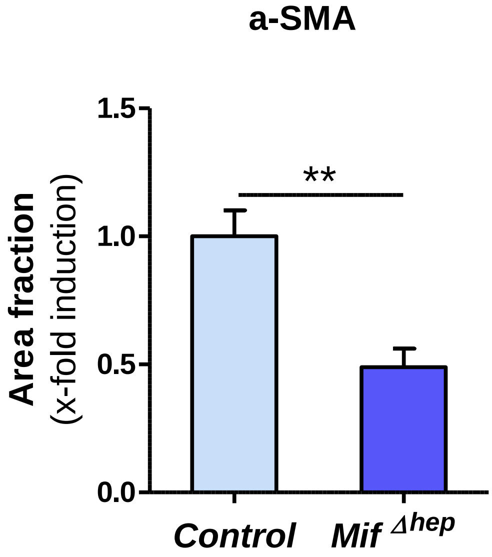

Supplementary Fig. 3. Quantification of a-SMA stainings from *Mif*$^{\Delta hep}$ (A) α-SMA stainings from 6 mice per group were analyzed by ImageJ quantification. Asterisks indicate statistical significance: *P<0.05; **P<0.01.

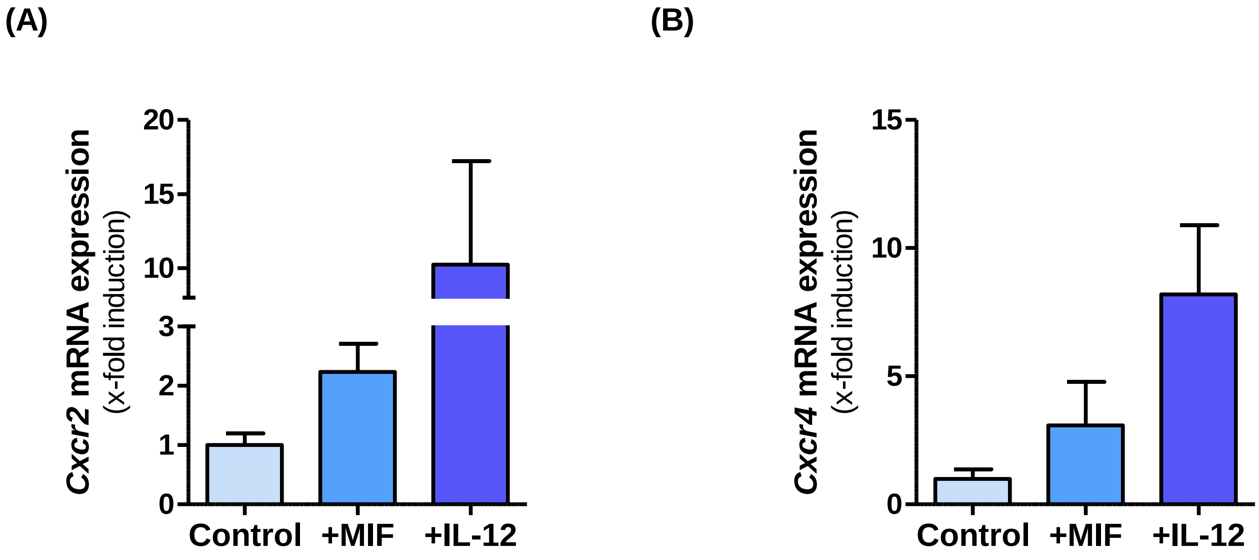

Supplementary Fig. 4. Expression patterns of MIF receptors *in vitro*. NKT cells were isolated from the spleen from untreated and 10 weeks old male *Mif*$^{-/-}$ mice. NKT cells were isolated via one negative and one positive selection with the MACS separator. Isolated cells were stimulated in vitro with 50 ng/ml of recombinant, murine MIF and as positive control with 20 pg/ml IL-12 for 24h. After stimulation mRNA analysis was performed to determine the expression levels of the (A) MIF receptor *Cxcr2* and (B) the MIF receptor *Cxcr4*.

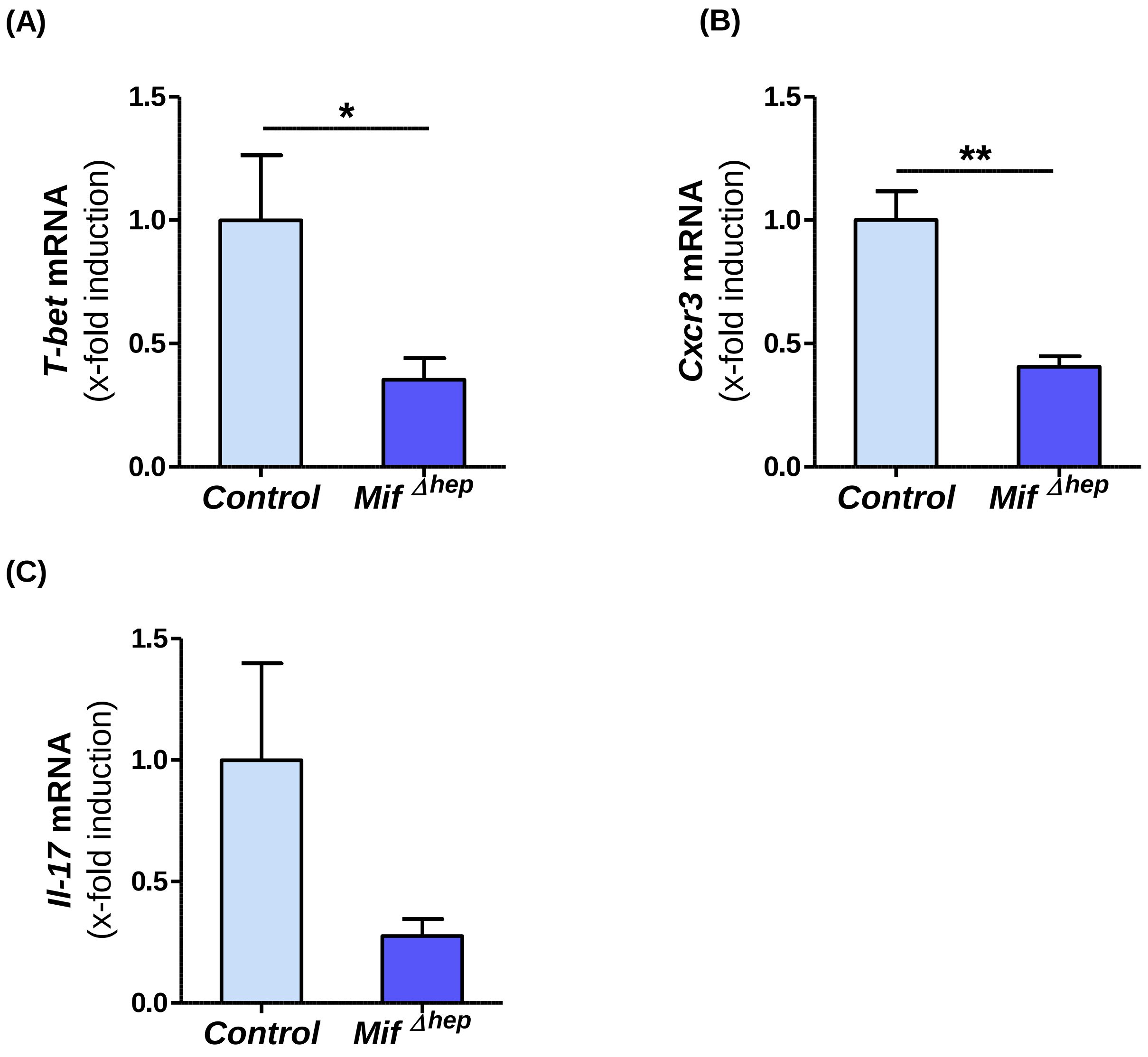

Supplementary Fig. 5. Expression patterns after eight weeks of MCD diet. (A) mRNA expression of *T-bet* after MCD diet for 8 weeks in *Mif*$^{\Delta hep}$ mice and littermates (n=6 per group). (B) mRNA expression of chemokine receptor *Cxcr3* in *Mif*$^{\Delta hep}$ mice and controls (n=6 per group). (C) mRNA expression of chemokine receptor *Il-17* in *Mif*$^{\Delta hep}$ mice and controls (n=6 per group). Asterisks indicate statistical significance: *P<0.05; **P<0.01.
