# Supplementary material for: Unexpected Pro-Fibrotic Effect of MIF in Non-Alcoholic Steatohepatitis Is Linked to a Shift in NKT Cell Populations
Source: Cells. 2021 Jan 28;10(2):252. doi: 10.3390/cells10020252 (PMC7918903; doi:10.3390/cells10020252)
Supplement: Supplementary file 1 [file cells-10-00252-s001.zip › cells-1068813-supplementary-PUB/Supplementary Tables.pdf]

Supplementary Table1. Clinical parameters of all patients (n = 22) at admission

**Parameters**	**Value**
Gender (female/male)	11/11
Age (in years)-median (range)	58.5 (33-80)
Fibrosis stage (Stage0/1/2/3/4)	9/8/2/2/1
Steatosis (% range)	16.1% (1% - 30%)
Bilirubin (mg/dl)	0.86 (0.21-5,21)
Alk. Phosphatase (U/L)	157.7 (54-508)
ALT (U/L)	40.3 (8-82)
AST (U/L)	36.8 (19-90)
γGT (U/L)	290.1 (23-2678)

Supplementary Table 2. Gating strategy

Neutrophils	**viable cells, single cells, CD45+, Ly6G+**
pDC	**viable cells, single cells, CD45+, Ly6G-, B220+, CD11c+**
cDC	**viable cells, single cells, CD45+, Ly6G-, B220-, CD11c+, MHCII+**
CD103+ cDC	**viable cells, single cells, CD45+, Ly6G-, B220-, CD11c+, MHCII+, CD103+**
CD11b+ cDC	**viable cells, single cells, CD45+, Ly6G-, B220-, CD11c+, MHCII+, CD11b+**
MoMacs	**viable cells, single cells, CD45+, Ly6G-, B220+, CD11c+, F4/80-**
$Ly6C^{hi}$	**viable cells, single cells, CD45+, Ly6G-, B220+, CD11c+, F4/80- $Ly6C^{high}$**
$Ly6C^{low}$	**viable cells, single cells, CD45+, Ly6G-, B220+, CD11c+, F4/80- $Ly6C^{low}$**
KC	**viable cells, single cells, CD45+, Ly6G-, B220+, CD11c+, F4/80+**
NK cells	**viable cells, single cells, CD45+, NK1.1+, CD3-**
NKT cells	**viable cells, single cells, CD45+, NK1.1+, CD3+**
T cells	**viable cells, single cells, CD45+, NK1.1-, CD3+**
CD4+ T cells	**viable cells, single cells, CD45+, NK1.1-, CD3+, CD4+, CD8-**
CD8+ T cells	**viable cells, single cells, CD45+, NK1.1-, CD3+, CD4-, CD8+**
B cells	**viable cells, single cells, CD45+, Ly6G-, B220+, Cd11c-**

Supplementary Table 3. Murine Primer used in this publication

Mif **forward**	5′-TTACCTGCACCGCTGTTCTTT-3′
Mif **reverse**	5′-TACCGGTGGATAAACACAGAACAC-3′
Col1a1 **forward**	5′-GGTTTCCACGTCTCACCATT-3′
Col1a1 **reverse**	5′-CGGCTCCTGCTCCTCTTAG-3′
Timp1 **forward**	5′-AGGTGGTCTCGTTGATTCGT-3′
Timp1 **reverse**	5′-GTAAGGCCTGTAGCTGTGCC-3′
Mmp2 **forward**	5′-GCAAGTTTCCGTTCCGCTTCC-3′
Mmp2 **reverse**	5′-CAGTACCAGTGTCAGTATCAG-3′
acta2 **forward**	5′-CTCTAGCACACAACTGTGAACGTTT-3′
acta2 **reverse**	5′-AAAGCTTTGGGCAGGAATGA-3′
Tgf-β **forward**	5′-GACCCTGCCATATTTGGA-3′
Tgf-β **reverse**	5′-GCCCGGGTTGTGTTGGT-3′
Il-2 **forward**	5′-GCCTAGAAGATGAACTTGGACCT-3′
Il-2 **reverse**	5′-GTTATTGAGGGCTTGTTGAGATG-3′
Il-4 **forward**	5′-AAACTCCATGCTTGAAGAAGAACT-3′
Il-4 **reverse**	5′-CTACGAGTAATCCATTTGCATGAT-3′
Il-13 **forward**	5′-TGGATTCCCTGACCAACATCTC-3′
Il-13 **reverse**	5′-GGTTACAGAGGCCATGCAATATC-3′
Tnf-α **forward**	5′-ACCACGCTCTTCTGTCTACTGA-3′
Tnf-α **reverse**	5′-TCCACTTGGTGGTTTGCTACG-3′
Fasl **forward**	5′-GTGGCCCATTTAACAGGGAACC-3′
Fasl **reverse**	5′-GGCTGGTTGTTGCAAGACTGAC-3′
Opn **forward**	5′-CTCCATCGTCATCATCATCG-3′

***Opn* reverse**	5′-TGCACCCAGATCCTATAGCC-3′
***Cd74* forward**	5′-GCTCCACCTAAAGTACTGACCAA-3′
***Cd74* reverse**	5′-GTGGCAAATAGTTACCGTTCTCG-3′
***Cxcr2* forward**	5′-GGTGGGGAGTTCGTGTAGAA-3′
***Cxcr2* reverse**	5′-CGAGGTGCTAGGATTTGAGC-3′
***Cxcr4* forward**	5′-TCAGTGGCTGACCTCCTCTT-3′
***Cxcr4* reverse**	5′-TTTCAGCCAGCAGTTTCCTT-3′
***Il-17* forward**	5′-TCAGCGTGTCCAAACACTGAG-3′
***Il-17* reverse**	5′-CGCCAAGGGAGTTAAAGACTT-3′
***T-bet* forward**	5′-AGCAAGGACGGCGAATGTT-3′
***T-bet* reverse**	5′-GGGTGGACATATAAGCGGTTC-3′
***Cxcr3* forward**	5′-AGAATCATCCTGGTCTGAGACAA-3′
***Cxcr3* reverse**	5′-AAGATAGGGCATGGCAGCTA-3′
***Vα14Jα18* forward**	5′-TCAAACAGGACACAGGCAAAGG-3′
***Vα14Jα18* reverse**	5′-TCCCTAAGGCTGAACCTCTATC-3′
***18sRNA* forward**	5′- GTAACCCGTTGAACCCCATT-3′
***18sRNA* reverse**	5′- CCATCCAATCGGTAGTAGCG-3′

Human Primer used in this publication

***MIF* forward**	5′-GCCGCGTTCATGTCGTAATA-3′
***MIF* reverse**	5′-GCGCCTGCGCATCAG-3′
***COL1a1* forward**	5′-GTCTTCTGGCCCCTCTGGTG-3′

***COL1a1* reverse**	5′-TCGCCCTGTTCGCCTGTCTCA-3′
***CD74* forward**	5′-ATGAGCAACTGCCCATGC-3′
***CD74* reverse**	5′-TACAGGAAGTAGGCGGTGGT-3′
***CXCR2* forward**	5′-CAGTTACAGCTCTACCCTGCC-3′
***CXCR2* reverse**	5′-CCAGGAGCAAGGACAGACCCC-3′
***CXCR4* forward**	5′-ACGGACAAGTACAGGCTGCAC-3′
***CXCR4* reverse**	5′-CCCAGAAGGGAAGCGTGA-3′
***ACTA2* forward**	5′-CCAGCTATGTGAAGAAGAAGAGG-3′
***ACTA2* reverse**	5′-GTGATCTCCTTCTGCATTCGGT-3′
***TGF-β* forward**	5′-CAATTCCTGGCGATACCTCAG-3′
***TGF-β* reverse**	5′-GCACAACTCCGGTGACATCAA-3′
***FASL* forward**	5′-GGATTGGGCCTGGGGATGTTTCA-3′
***FASL* reverse**	5′-TTGTGGCTCAGGGGCAGGTTGTTG-3′
***Opn* forward**	5′-TTGCAGCCTTCTCAGGCAA-3′
***Opn* reverse**	5′-GGAGGCAAAAGCAAATCACTG-3′
***T-bet* forward**	5′-GTCCAACAATGTGACCCAGAT-3′
***T-bet* reverse**	5′-ACCTCAACGATATGCAGCCG-3′
***Cxcr3* forward**	5′-ACCCAGCAGCCAGAGCACC-3′
***Cxcr3* reverse**	5′-TCATAGGAAGAGCTGAAGTTCTCCA-3′
***INF-γ* forward**	5′-AGGGAAGCGAAAAAGGAGTCA-3′
***INF-γ* reverse**	5′-GGACAACCATTACTGGGATGCT-3′
***Vα24Jα18* forward**	5′-CTGGAGGGAAAGAACTGC-3′
***Vα24Jα18* reverse**	5′-TGTCAGGGAAACAGGACC-3′
***18sRNA* forward**	5′- GTAACCCGTTGAACCCCATT-3′

***18sRNA* reverse**	5′- CCATCCAATCGGTAGTAGCG-3′

Supplementary Table 4. Summary of correlation analysis of intrahepatic mRNA expression level of *MIF*, its receptors *CXCR2*/*CXCR4*/*CD74*, NKT cell marker and Fibrosis-associated genes in NAFLD patients' samples as assessed by qRT-PCR analysis. 22 human samples with different stages of steatosis and fibrosis were analyzed. Red

	MIF	***CD74***	***CXCR2***	***CXCR4***	***ATAC2***	***COL1a1***	***TGFß***	***FASL***	***OPN***	***INFy***
MIF					P = 0.3021<br>R = 0.2429	P = 0.0053<br>R = 0.729	P = 0.171<br>R = 0.5263	P = 0.0509<br>R = 0.4313	P = 0.1233<br>R = 0.3659	P = 0.8416<br>R = 0.0464
CD74					P = 0.008<br>R = 0.577	P = 0.6174<br>R = 0.1157	P< 0.001<br>R = 0.9689	P = 0.0097<br>R = 0.5509	P = 0.0035<br>R = 0.6347	P = 0.0003<br>R = 0.7237
CXCR2					P = 0.0017<br>R = 0.8096	P = 0.2682<br>R = 0.2532	P = 0.0002<br>R = 0.8598	P = 0.0015<br>R = 0.6465	P = 0.0915<br>R = 0.3979	P< 0.001<br>R = 0,9406
CXCR4					P< 0.001<br>R = 0.7509	P = 0.0714<br>R = 0.0889	P< 0.001<br>R = 0.8497	P = 0.0061<br>R = 0.5774	P = 0.0178<br>R = 0.5366	P = 0.0006<br>R = 0.699
ATAC2	P = 0.3021<br>R = 0.2429	P = 0.008<br>R =0. 577	P = 0.0017<br>R = 0.8096	P< 0.001<br>R = 0.7509				P = 0.0009<br>R = 0.8338	P = 0.0046<br>R = 0.8069	P = 0.0007<br>R = 0.8421
COL1a1	P = 0.0053<br>R = 0.729	P = 0.6174<br>R = 0.1157	P = 0.2682<br>R = 0.2532	P = 0.0714<br>R = 0.0889				P = 0.5693<br>R = 0.7545	P = 0.4127<br>R = 0.1995	P = 0.4201<br>R = 0.1908
TGFß	P = 0.171<br>R = 0.5263	P< 0.001<br>R = 0.9689	P = 0.0002<br>R = 0.8598	P< 0.001<br>R = 0.8497				P = 0.0357<br>R = 0.6958	P < 0.0001<br>R = 0.9059	P < 0.0001<br>R = 0.9039
FASL	P = 0.0509<br>R = 0.4313	P = 0.0097<br>R = 0.5509	P = 0.0015<br>R = 0.6465	P = 0.0061<br>R = 0.5774	P = 0.0009<br>R = 0.8338	P = 0.5693<br>R = 0.7545	P = 0.0357<br>R = 0.6958			
OPN	P = 0.1233<br>R = 0.3659	P = 0.0035<br>R = 0.6347	P = 0.0915<br>R = 0.3979	P = 0.0178<br>R = 0.5366	P = 0.0046<br>R = 0.8069	P = 0.4127<br>R = 0.1995	P < 0.0001<br>R = 0.9059			
INFy	P = 0.8416<br>R = 0.0464	P = 0.0003<br>R = 0.7237	P< 0.001<br>R = 0.9406	P = 0.0006<br>R = 0.699	P = 0.0007<br>R = 0.8421	P = 0.4201<br>R = 0.908	P < 0.0001<br>R = 0.9039			
